# Antitumor activity of the polo-like kinase inhibitor, TAK-960, against preclinical models of colorectal cancer

**DOI:** 10.1186/s12885-018-4036-z

**Published:** 2018-02-05

**Authors:** Peter J. Klauck, Stacey M. Bagby, Anna Capasso, Erica L. Bradshaw-Pierce, Heather M. Selby, Anna Spreafico, John J. Tentler, Aik Choon Tan, Jihye Kim, John J. Arcaroli, Alicia Purkey, Wells A. Messersmith, Keisuke Kuida, S. Gail Eckhardt, Todd M. Pitts

**Affiliations:** 1 Division of Medical Oncology, School of Medicine, University of Colorado, Anschutz Medical Campus, Aurora, CO USA; 2 Department of Pharmaceutical Sciences, Skaggs School of Pharmacy and Pharmaceutical Sciences, University of Colorado, Anschutz Medical Campus, Aurora, CO USA; 3 University of Colorado Cancer Center, University of Colorado, Anschutz Medical Campus, Aurora, CO USA; 4 Millennium Pharmaceuticals, Inc., a wholly owned subsidiary of Takeda Pharmaceutical Company Limited, Cambridge, MA USA; 5 Takeda California, San Diego, CA USA

**Keywords:** TAK-960, Plk1, Colorectal cancer, Patient-derived xenograft

## Abstract

**Background:**

Polo-like kinase 1 (Plk1) is a serine/threonine kinase that is a key regulator of multiple stages of mitotic progression. Plk1 is upregulated in many tumor types including colorectal cancer (CRC) and portends a poor prognosis. TAK-960 is an ATP-competitive Plk1 inhibitor that has demonstrated efficacy across a broad range of cancer cell lines, including CRC. In this study, we investigated the activity of TAK-960 against a large collection of CRC models including 55 cell lines and 18 patient-derived xenografts.

**Methods:**

Fifty-five CRC cell lines and 18 PDX models were exposed to TAK-960 and evaluated for proliferation (IC_50_) and Tumor Growth Inhibition Index, respectively. Additionally, 2 KRAS wild type and 2 KRAS mutant PDX models were treated with TAK-960 as single agent or in combination with cetuximab or irinotecan. TAK-960 mechanism of action was elucidated through immunoblotting and cell cycle analysis.

**Results:**

CRC cell lines demonstrated a variable anti-proliferative response to TAK-960 with IC_50_ values ranging from 0.001 to > 0.75 μmol/L. Anti-proliferative effects were sustained after removal of drug. Following TAK-960 treatment a highly variable accumulation of mitotic (indicating cell cycle arrest) and apoptotic markers was observed. Cell cycle analysis demonstrated that TAK-960 treatment induced G2/M arrest and polyploidy. Six out of the eighteen PDX models responded to single agent TAK-960 therapy (TGII< 20). The addition of TAK-960 to standard of care chemotherapy resulted in largely additive antitumor effects.

**Conclusion:**

TAK-960 is an active anti-proliferative agent against CRC cell lines and PDX models. Collectively, these data suggest that TAK-960 may be of therapeutic benefit alone or in combination with other agents, although future work should focus on the development of predictive biomarkers and hypothesis-driven rational combinations.

**Electronic supplementary material:**

The online version of this article (10.1186/s12885-018-4036-z) contains supplementary material, which is available to authorized users.

## Background

Polo-like kinase 1 (Plk1) is a highly-conserved serine/threonine kinase crucial to the regulation of mitosis. An essential gene, Plk1 functions to initiate mitosis, control progression through M phase and to trigger mitotic exit. Plk1 expression begins in late S phase, reaching peak activity during late G_2_ and early M initiating mitosis by phosphorylating targets cyclin B1 and Cdc25c [1, 2]. During mitosis, Plk1 localizes to centrosomes, the equatorial spindle midzone, kinetochore and centromere region and the post-mitotic bridge [3–5]. Considering Plk1’s broad involvement in mitotic machinery, it is not surprising that Plk1 is central to the metaphase-anaphase transition and mitotic exit. Plk1 is involved in centrosome maturation, kinetochore assembly, spindle formation (including the spindle activation checkpoint), activation of the anaphase promoting complex, chromosome segregation and cytokinesis [1–3]. At mitotic exit APC/C–CDH1 ubiquitinates Plk1, triggering proteasomal degradation [6].

When DNA damage is detected, there are many cellular responses that coordinate cell cycle arrest to allow DNA to be repaired. Plk1 is a target for several of these redundant mechanisms. Double strand DNA breaks trigger activation of ATM/Chk2 pathway leading to dephosphorlyation and inactivation of Plk1 [7]. If DNA damage occurs, but Plk1 has yet to be activated, ATM/ATR prevents Plk1 activation by triggering the degradation of the Plk1 activator Bora through a phosphorylation event [8]. In a parallel (ATM independent) pathway, double strand breaks (DSB) activate the canonical (proteasomal) Plk1 degradation pathway. DSBs activate Cdh14B, which in turn activate APC/C^cdh1^ causing proteasomal degradation of Plk1 to maintain the G2/M checkpoint [9]. Concurrent with Plk1 inactivation, DNA damage activates tumor suppressor p53 through the ATM/Chk2/p53 signaling pathway. p53, known as the guardian of the genome, plays a major role in DNA repair and genomic stability. Activated p53 upregulates transcription of downstream effectors that mediate cellular processes for repairing DNA, arresting the cell cycle, and triggering apoptosis [10].

In addition to their inverse regulation by ATM/ATR, Plk1 and p53 reciprocally regulate each other. Directly, activated Plk1 directly binds to the DNA binding domain of p53, inhibiting its transactivation activity [11]. Indirectly, Plk1 phosphorylates MDM2, stimulating MDM2-mediated turnover of p53 [12]. p53 regulates Plk1 expression by localizing to the PLK1 gene promoter, binding E2F1 and decreasing PLK1 transcription [13]. Indirectly, the downstream effector of p53, p21/waf1 inhibits Plk1 expression by targeting specific sequences in the promoter [2, 14]. Through cross regulation, p53 and Plk1 directly contribute in the regulation of stop/go cell cycle decision.

The elegant, but redundant regulation of Plk1 and p53 by ATM/ATR, as well as, reciprocal regulation by each other has been implicated in tumorigenesis. p53 is the most commonly mutated gene across all cancers and is mutated in 40–50% of colorectal cancers [15, 16]. p53 mutations are thought to play a major role in carcinogenesis [17]. PLK1 has been found to be upregulated in many tumor types including: melanoma, non-small-cell lung, prostate, and colorectal and overexpression of Plk1 correlates with a poor clinical prognosis [1, 18–21]. It is thought that overexpression of Plk1 leads to genomic instability by enabling cells to down-regulate p53 and override cell cycle checkpoints [2, 20, 22]. Plk1 regulators Cdc14B and APC/C^cdh1^ have been found to be downregulated in several tumor types including prostate and brain cancer [9].

These data have drawn attention to the development of anti-Plk1 therapeutics. Inhibition of Plk1 by siRNA or small molecule inhibitors has resulted in cell cycle arrest in metaphase and the induction of apoptosis in cancer cell lines [18, 23]. Several Plk1 inhibitors are currently under preclinical and clinical development [24]. Indeed, the Plk1 inhibitor volasertib (BI-6727) recently obtained FDA breakthrough therapy designation for the treatment of acute myeloid leukemia.

TAK-960 is a recently discovered ATP-competitive inhibitor of Plk1. It is orally available and Plk1 selective. TAK-960 has subnanomolar activity (IC_50_ 0.8 nmol/L) against Plk1 compared to other Plk family members (IC_50_ Plk2 16.9 nmol/L, Plk3 50.2 nmol/L) [25]. Plk1 inhibition by TAK-960 has been shown to lead to G_2_-M phase mitotic arrest and display the characteristic monopolar spindle morphology and aberrant spindle accumulation described in other Plk1 inhibitors [18, 26, 27]. TAK-960 has demonstrated robust antitumor activity in cell line xenograft models of several tumor types with favorable drug tolerability and PK/PD profiles [18].

In the current study, we investigated the efficacy of TAK-960 against a large panel of well-characterized colorectal cancer models. Since numerous phase I clinical trials have shown small molecule inhibitors have limited efficacy when administered as a single agent, we also investigated the efficacy of TAK-960 in combination with standard agents for both KRAS^WT^ and KRAS^MT^ colorectal cancer models [28–30].

## Methods

### Compounds and reagents

TAK-960 [4-[(9-cyclopentyl-7,7-difluoro-5-methyl-6-oxo-6,7,8,9-tetrahydro-5H-pyrimido[4,5-b][1,4]diazepin-2-yl)amino]-2-fluoro-5-methoxy-N-(1-methylpiperidin-4-yl) benzamide] was provided by Millennium, The Takeda Oncology Company (Cambridge, MA). All antibodies were obtained from Cell Signaling Technologies (Danvers, MA).

### Cell lines and culture

Human colorectal cancer cell lines were obtained from ATCC (Manassas, VA, USA), DSMZ Cell Line Bank (Braunschweig, Germany), ECACC (Sigma, St. Louis, MO) and the Korean Cell Line Bank (KCLB) (Seoul, South Korea). The GEO cell line was a generous gift from Dr. Fortunato Ciardiello (Cattedra di Oncologia Medica, Dipartimento Medico-Chirurgico di Internistica Clinica e Sperimentale “F Magrassi e A Lanzara,” Seconda Università degli Studi di Napoli, Naples, Italy). KM20 were a generous gift from Dr. Scott Kopetz from MD Anderson Cancer Center, Houston, TX, USA. The 55 human colorectal cancer cell lines used in this study were: CL-11(DSMZ ACC 467), CL-34(DSMZ ACC520), COLO201 (ATCC® CCL-224™), COLO205 (ATCC® CCL-222™), COLO678 (DSMZ ACC 194), DLD1 (ATCC® CCL-221™), GP2D (SIGMA 95090714), GP5D (SIGMA 95090715), HCA-24 (SIGMA 06061903), HCA-46 (SIGMA 07031601), HCA7 (SIGMA 06061902), HCT116 (ATCC® CCL-247™), HCT15 (ATCC® CCL-225™), HCT8 (ATCC® CCL-244™), HT15 (SIGMA 85061104), HT29 (ATCC® HTB-38™), HT55 (SIGMA 85061105), LOVO (ATCC® CCL-229™), LS1034 (ATCC® CRL-2158™), LS123 (ATCC® CCL-255™), LS174T (ATCC® CL-188™), LS180 (ATCC® CL-187™), LS513 (ATCC® CRL-2134™), MDST8 (SIGMA 99011801), Mip101 (ECACC CVCL-H689), NCI-H508 (ATCC® CCL-253™), NCI-H716 (ATCC® CCL-251™), NCI-H747 (ATCC® CCL-252™), RKO (ATCC® CRL-2577™), SKCO1 (ATCC® HTB-39™), SNU-1235 (KCLB 01235.1), SNU-1411 (KCLB 01411.1), SNU-1544 (KCLB 01544.1), SNU-1684 (KCLB 01684), SNU-1746 (KCLB 01746), SNU-254 (KCLB 00254), SNU-70 (KCLB 00070), SNU-796 (KCLB 00796.1), SNU-977 (KCLB 00977.1), SNU-C1 (KCLB 0000C1), SNU-C2B (KCLB 0000C2B), SNU1460 (KCLB 01460.1), SW1116 (ATCC® CCL-233™), SW1417 (ATCC® CCL-238™), SW1463 (ATCC® CCL-234™), SW403 (ATCC® CCL-230™), SW48 (ATCC® CCL-231™), SW480 (ATCC® CCL-228™), SW620 (ATCC® CCL-227™), SW837 (ATCC® CCL-235™), SW948 (ATCC® CCL-237™), T84 (ATCC® CCL-248™), WiDr (ATCC® CCL-218™). All cell lines were cultured in RPMI media supplemented with 10% fetal bovine serum, 1% penicillin-streptomycin and 1% MEM nonessential amino acids and routinely screened for the presence of mycoplasma (MycoAlert, Cambrex Bio Science, Baltimore, MD, USA). Cell lines were maintained at 37 °C with 5% CO_2_.

### Cell proliferation (CyQuant)

Cell proliferation was assessed using CyQuant assay (Life Technologies, Carlsbad CA). Variable numbers of cells, relative to their logarithmic growth phase, were suspended in 200uL of media and plated in sterile 96 well black wall plates. Plates were incubated for 24 h to allow cells to attach. Cell lines were exposed to TAK-960 at increasing concentrations (0-.75 μmol/L) for 72 h. After 72 h dye/lysis buffer was added to the plate and fluorescence was measured on a Synergy 2 microplate reader (Biotek, Winooski, VT). IC_50_ was calculated from at least 3 independent experiments for each cell line. Each bar represents this mean IC_50_ +/- SEM and corresponds to the matrix of mutational status of KRAS, BRAF and PIK3CA, p53, and APC.

### Immunoblotting

CRC cell lines were seeded in 6-well plates (density determined for each cell line based on growth rate) as previously described [31]. The following day, cells were treated with increasing concentrations of TAK-960 (0.01, 0.05, 0.1, 0.2, 0.5, and 1 μM) or mock treated control for 8, 24, 48, and 72 h. Following exposure, media from each well was collected and centrifuged for 5 min at 1200 rpm, supernatant was removed and cell pellet was mixed with adherent cells previously scraped into RIPA buffer containing protease and phosphatase inhibitors (Pierce, Santa Ana, CA). Cells were lysed with a Qsonica Q55 probe sonicator for 20 s x2 (Qsonica, Newtown, CT). Samples were centrifuged at 16,000 g at 4 °C for 10 min. Total protein was determined using the Pierce 660 nm Protein Assay, (Pierce, Santa Ana, CA). Fifty micrograms of protein were electrophoresed on 4–12% Bis-Tris precast gels (Life Technologies, Carlsbad, CA) and transferred to nitrocellulose membrane using Pierce G2 Fast Blotter (Pierce, Santa Ana, CA). Membranes were blocked for 1 h in blocking buffer (0.1% Casein solution in 0.2X PBS) at room temperature. Membranes were incubated overnight at 4 °C in blocking buffer plus 0.1% Tween-20 with the following primary antibodies at 1:1000 dilutions: pPlk1, Plk1, caspase 3, PARP, cyclin B1, p53, Bcl-xl, pHH3, and β-actin. Blots were washed 3 × 10 min in 1X TBS containing 0.1% Tween-20 and incubated with the appropriate secondary goat anti-rabbit and goat anti-mouse immunoglobulin G (H + L) DyLight conjugated antibodies (Cell Signaling, Danvers, MA) at a 1:15,000 dilution for 1 h at room temperature. Blots were washed 3 × 15 min and then developed using the Odyssey Infrared Imaging System (LI-COR, Lincoln, NE).

### Clonogenic colony formation assay

CRC cell lines were seeded in 6-well plates (2–20,000 cells/well depending on individual cell line growth rate). The following day cell lines were exposed to TAK-960 (0.1, 0.5, 1 and 2 μM) or mock treated control for 72 h. After 72 h of drug exposure, drug containing media was removed, each well was washed with 1 mL PBS and media containing no drug was added for an additional 72 h for regrowth. After the 72 h regrowth phase, cells were fixed with 100% methanol and stained with 1X crystal violet for 30 min. Methanol and crystal violet was removed and each well was washed three times with water and allowed to air dry. Colony area was quantified using Image J (Colony Area Plugin) [32].

### Cell cycle analysis

CRC cell lines were seeded in 6-well plates (density determined for each cell line based on growth rate). The following day, cell lines were treated with one of two concentrations of TAK-960 (0.1 and 1 μM) or mock treated control for 24 and 48 h. Cells were trypsinized, washed in PBS + 2% FBS and re-suspended in Krishan’s stain, incubated overnight at 4 °C and analyzed for cell cycle and ploidy using flow cytometry by the University of Colorado Cancer Center Flow Cytometry Core Facility.

### Patient derived xenograft models

Female athymic nude (nu/nu) mice were purchased from Harlan Laboratories (Indianapolis, IN). Approximately 3 mm^3^ tumor sections were injected subcutaneous into both flanks of the mice. Tumors were injected into 5–6 mice (at least 10 evaluable tumors) per group. When tumor volumes reached ~ 150–200 mm^3^ the mice were randomized into either vehicle or TAK-960 groups. Mice were treated daily with TAK-960 (10 mg/kg) or vehicle control daily by oral gavage for at least 28 days. Mice were monitored daily for signs of toxicity. Tumor size was evaluated twice per week by caliper measurements using the following equation: tumor volume = (length × width^2^) × 0.52 and recorded in the Study Director Program (South San Francisco, CA). Tumor growth inhibition index was calculated from average volume of the treated (*V*_t_) and vehicle control (*V*_vc_) groups, with the equation: TGII (%) = (*V*_t final_ -*V*_t initial_)/(*V*_vc final_ -*V*_vc intial_) × 100. Therefore, if TAK-960 treatment resulted in no change in growth vs vehicle treated controls, TGII (%) =100. If TAK-960 treatment results in 80% tumor growth compared to vehicle treated control tumors, TGII (%) < 20. For combination studies, mice were randomized as above into 4 groups and were treated with either vehicle, TAK-960 (5 mg/kg) daily, cetuximab (KRAS^WT,^ 400 μg/mouse) twice weekly, irinotecan (KRAS^MT^ 15 mg/kg) once weekly, or the combination for at least 20 days. Tumor measurements were obtained as above.

### Statistical analysis

Results from clonogenic colony formation and cell cycle analysis assays were analyzed for statistical significance with GraphPad Prism V5.04 software using paired and unpaired T-Tests, respectively. * indicates *p* < 0.05 and ** indicates *p* < 0.01. In the cell cycle analysis assay ## is used to indicate a significance of p < 0.01 for the aneuploid (*N* > 4) cell population.

## Results

### TAK-960 is a potent antiproliferative agent against colorectal cancer cell lines

**Fig. 1:**
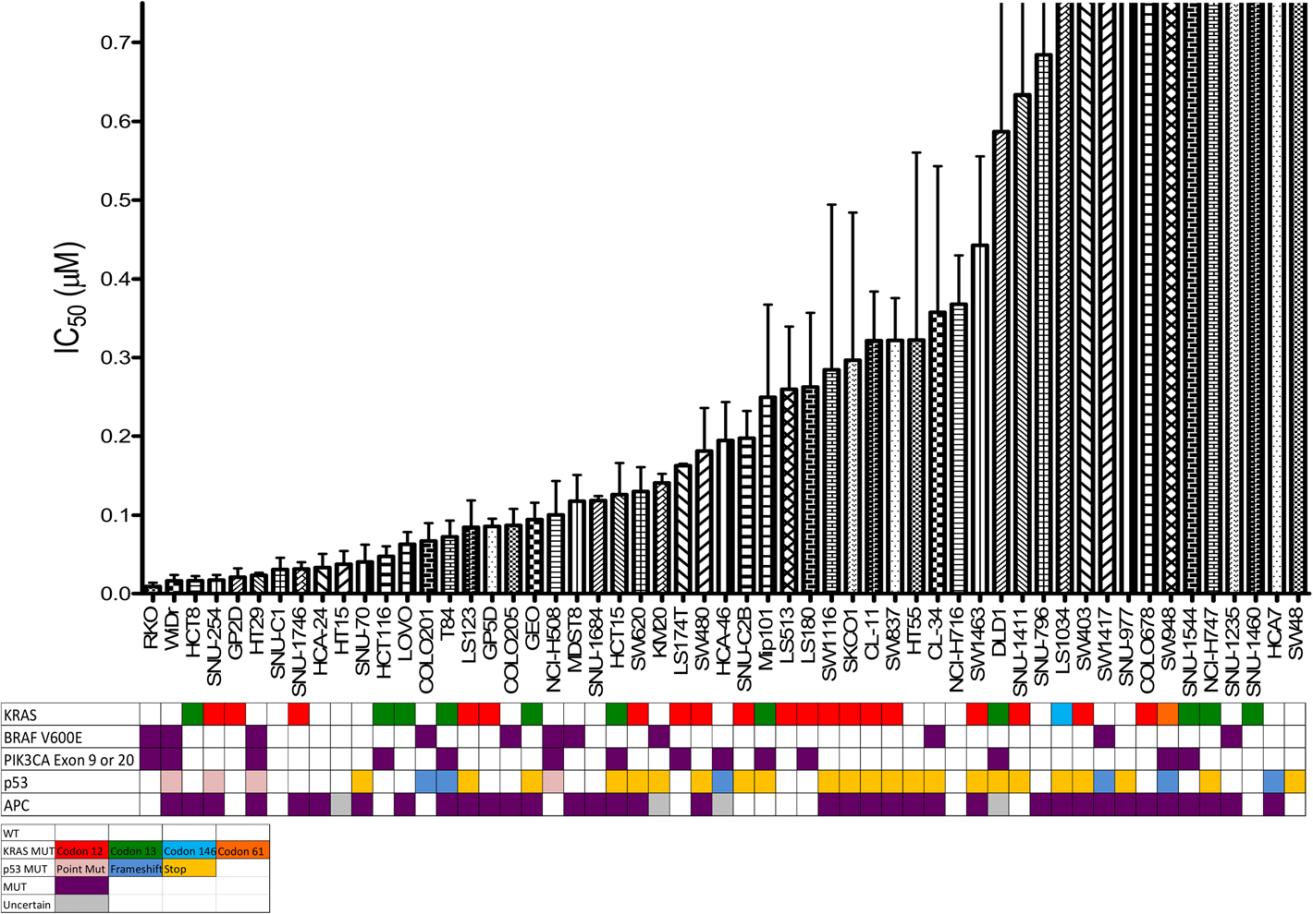
The effects of TAK-960 on CRC cell lines in vitro*.* Fifty-five CRC cell lines were exposed to increasing concentrations of TAK-960. Proliferation was assessed by CyQuant Assay. IC_50_ +/- SEM was calculated for all cell lines and ranged from < 0.001 μM to > 0.75 μM. Mutant genes are shown by colored boxes. There is no significant correlation between sensitivity and the genetic mutations depicted. N≥3

The anti-proliferative effects of TAK-960 were assessed across a panel of 55 colorectal cell lines (Fig. 1 and Additional file 1: Table S1). Cell lines were exposed to 7 different concentrations of TAK-960 for 72 h. The relative amount of DNA was measured using a CyQuant proliferation assay and the results were normalized to the mock treated control. There were variable anti-proliferative responses with IC_50_ values ranging from less than 0.001 μM to greater than 0.75 μM. Mutations in KRAS, BRAF, PIK3CA, p53, or APC did not correlate with response to TAK-960 (Fig. 1). Two responsive and two non-responsive CRC cell lines were selected for further pharmacodynamic analyses to elucidate the mechanism of action. Cell lines were selected based on IC_50_ as well as mutational status to incorporate common CRC mutations. Responsive cell lines chosen were WIDR (KRAS^WT^ /BRAF^V600E^/PIK3CA^P449T^) and HCT116 (KRAS^G13D^/PIK3CA^H1047R^). Non-responsive cell lines chosen were DLD1 (KRAS^G13D^/ PIK3CA^D549N E545K^) and COLO678 (KRAS^G12D^/PIK3CA^WT^).

### TAK-960 inhibits colony growth and regrowth

**Fig. 2:**
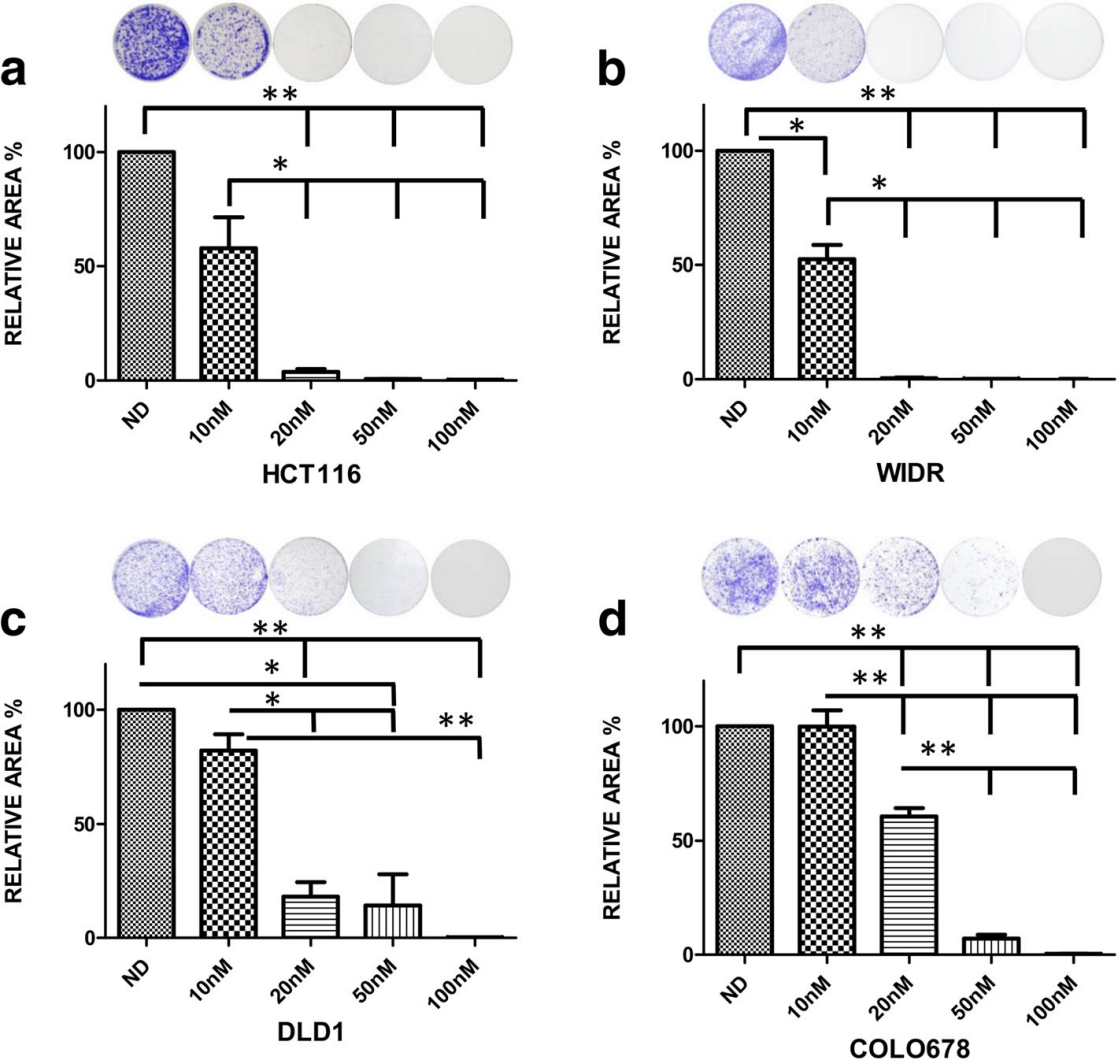
Clonogenic analysis of four CRC cell lines exposed to TAK-960. **a** HCT116, **b** WIDR, **c** DLD1, **d** COLO678 were plated in 6 well plates and exposed to increasing concentrations of TAK-960 for 72 h or mock treated control. Drug was removed and replaced with media to allow for regrowth of clones. Cells were stained with crystal violet, photographed and quantitated using ImageJ software using the Colony Area Plugin. *p < 0.05, **p < 0.01 by paired t-test. N≥3

To assess TAK-960 treatment effects on colony formation and growth, clonogenic assays were performed on the two responsive and two non-responsive cell lines selected for further experiments. As compared to the untreated control, TAK-960 treatment of CRC cell lines HCT116, WIDR, DLD1 and COLO678 decreased colony formation, dose dependently (*p* < 0.05) with little to no visible colonies in doses of TAK-960 greater than 10 nM (Fig. 2a, b). The decrease in colony growth was evaluated qualitatively by a visual decrease in crystal violet stain and quantitatively by Image J (ColonyArea plugin). Colony formation of CRC cell lines DLD1 and COLO678 decreased dose dependently, however, < 10% area coverage occurred at TAK-960 levels greater than 20 nM. In all CRC cell lines, there was no observed regrowth of cell colonies in the 100 nM TAK-960 treatment wells once TAK-960 was removed (Fig. 2).

### Immunoblot analysis of TAK-960 mechanism of action

**Fig. 3:**
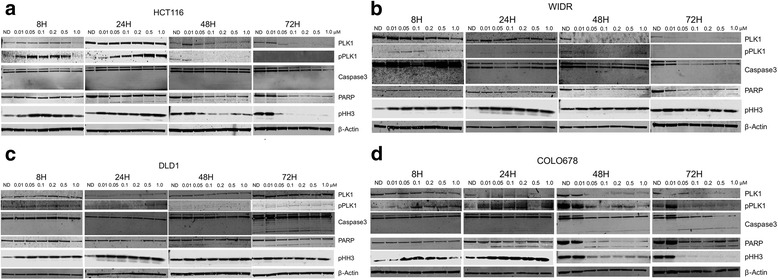
Pharmacodynamic effects of TAK-960 on relevant downstream effectors. Two sensitive (**a, b**) and two resistant (**c, d**) CRC cell lines were exposed to TAK-960 (0.01, 0.05, 0.1, 0.2, 0.5, 1.0 μM) or mock treated control for 8, 24, 48 h, and 72 h. *N* = 3

Immunoblotting was performed to elucidate the mechanism of action of TAK-960 in the four CRC cell lines. CRC cell lines were treated with increasing concentrations of TAK-960 (0.5–1 μM) for 8, 24, 48, and 72 h (Fig. 3). Recent studies have shown Plk1 and Phospho-Plk1 accumulation upon exposure to Plk1 inhibition by TAK-960 and CBB2001, respectively [33, 34]. At early time points, Plk1 levels remained constant in both sensitive and resistant cell lines. At later time points (> 24 h for HCT116 and WIDR, > 8 h for COLO678) levels of Plk1 declined when CRC cell lines were treated with TAK-960. Only the resistant DLD1 CRC cell line had constant levels of Plk1 at all doses and time points. Interestingly, Phospho-Plk1 induction was observed at early time points (< 48 h) in HCT116 and WIDR and COLO678. At later time points, P-Plk1 was reduced when exposed to TAK-960 in all three of these cell lines. P-Plk1 levels did not change in more resistant CRC cell line DLD1 either with time or higher concentrations of TAK-960.

Histone H3 becomes phosphorylated during chromosome condensation in mitosis and meiosis. Following a complete cellular division, histone H3 is dephosphorylated [35]. Accumulation of pHH3 was observed after TAK-960 exposure in both sensitive (HCT116 and WIDR) and resistant (DLD1 and COLO678) CRC cell lines, however, this accumulation occurred only at early time points (8 and 24 h). An increase in pHH3 was not seen at 48 and 72 h time points. Prolonged exposure (> 24 h) to TAK-960 treatment reduced pHH3 levels in HCT116 and COLO678, whereas, pHH3 levels were sustained in WIDR and DLD1.

Similar to pHH3, cyclin B1 accumulates during mitosis, peaking at the G2-M interface. During the anaphase step of mitosis, cyclin B1 is rapidly degraded by APC [36, 37]. Accumulation of cyclin B1 is indicative of G2/M cell cycle arrest [37, 38]. Cyclin B1 levels were variable with no appreciable pattern to cyclin B1 expression in any of the CRC cell lines treated with TAK-960 (data not shown).

Presence of caspase 3 and PARP cleavage products are well-established markers of apoptosis [39–41]. Caspase 3 cleavage was only observed in the more resistant CRC cell lines and even then, only at later time points, being most visible at 72 h. While levels of PARP cleavage product were variable over the time course of TAK-960 exposure between CRC cell lines, common in all four cell lines was the absence at 8 h followed by detection at 72 h. It has been previously established that Bcl family proteins, including BCL-XL have anti-apoptotic and anti-proliferative functions as well as to cause cell cycle arrest and delay cell cycle entry [42, 43]. There were no observable changes in BCL-XL levels due to an increase in duration or levels of TAK-960 exposure as compared to untreated controls (data not shown).

As mentioned above, tumor suppressor p53 and Plk1 function to counterbalance each other in the cell, contributing in opposite action to arrest and progress the cell cycle, respectively. There were no appreciable changes in p53 levels in all CRC cell lines with length or concentration of TAK-960 exposure (data not shown).

Levels of β-actin (loading control) were consistent across TAK-960 exposure duration and dose.

### TAK-960 treatment results in G2/M cell cycle arrest and polyploidy

**Fig. 4:**
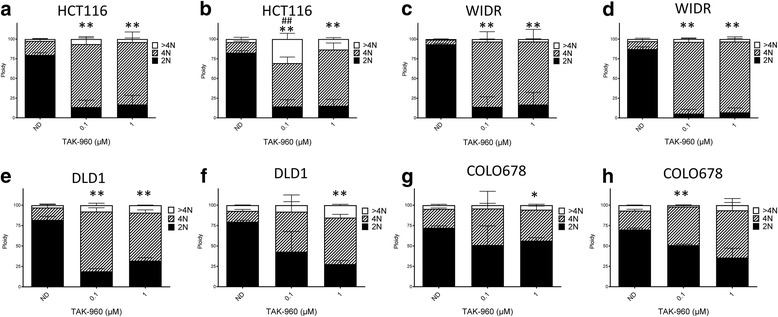
Cell cycle/ploidy analysis on two sensitive and two resistant CRC cell lines. Cells were exposed to TAK-960 (0.1 and 1 μM) or mock treated control for 24 and 48 h, stained with Krishan’s and analyzed for ploidy by flow cytometry. **a**, **b** HCT116, 24 and 48 h respectively, **c**, **d** WIDR, 24 and 48 h respectively, **e**, **f** DLD1, 24 and 48 h respectively, **g**, **h** COLO678, 24 and 48 h respectively. 4 N * *p* < 0.05, **p < 0.01 > 4 N, ## *p* < 0.01 by unpaired t-test. *N* = 3

Flow cytometry was utilized to determine the effects of TAK-960 on cell cycle dynamics. The four CRC cell lines were treated with two concentrations of TAK-960 (0.1 and 1μM) for 24 and 48 h (Fig. 4). Upon exposing HCT116 and WIDR to both 0.1 and 1μM of TAK-960, the proportion of diploid cells at 24 h decreased (Fig. 4a, c). This reduction was sustained at the 48 h time point (Fig. 4b, d). Correspondingly, the proportion of tetraploid cells, indicating a G2/M arrest, significantly increased (*p* < 0.01) under all treated conditions. Interestingly, in HCT116 cells there was a dramatic increase in the percentage of aneuploid (> 4 N) cells at the 0.1 μM concentration (p < 0.01) of TAK-960 at 48 h that was not observed in the WIDR cells (Fig. 4d). DLD1 (Fig. 4e, f) also demonstrated an increase in tetraploid cells (p < 0.01), however, there was not as dramatic of a reduction in COLO678 (Fig. 4g, h). In DLD1 and COLO670, an increase in the aneuploid cell population did not occur.

### TAK-960 is active against colorectal cancer patient derived xenograft models

**Fig. 5:**
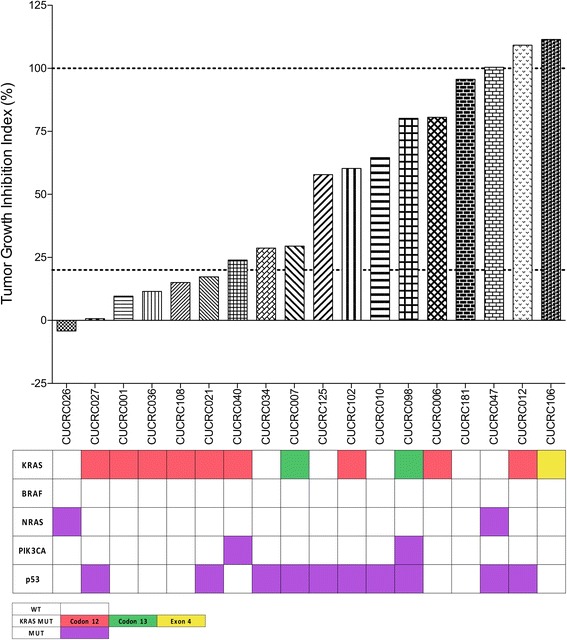
Antitumor Activity of TAK-960 as Measured by Tumor Growth Inhibition Index (TGII) in CRC Patient-Derived Tumor Xenograft Models (PDX). TGII = treated over control, thus lower numbers indicate greater tumor response. 6/18 (33%) models were considered sensitive with TGII < 20. Genetic mutations are indicated by colored boxes.

To investigate the effectiveness of TAK-960 in vivo, 18 colorectal cancer patient-derived xenograft models (PDX) were treated with TAK-960 for at least 28 days. PDX models were treated daily (QD) with 10 mg/kg of TAK-960. Response to TAK-960 varied among PDX models with tumor growth inhibition indices (TGII) ranging from − 4.17 to 111.48 (Fig. 5). Six PDX models were considered responsive (TGII< 20), one of which (CUCRC026) demonstrated minor tumor regression (TGII< 0). The presence of KRAS, BRAF, NRAS, PIK3CA, or p53 mutations did not correlate with PDX responsiveness to TAK-960. Weight loss and other indicators of drug toxicity were not observed in mice treated with TAK-960.

### TAK-960 in combination with standard agents in CRC PDX models

**Fig. 6:**
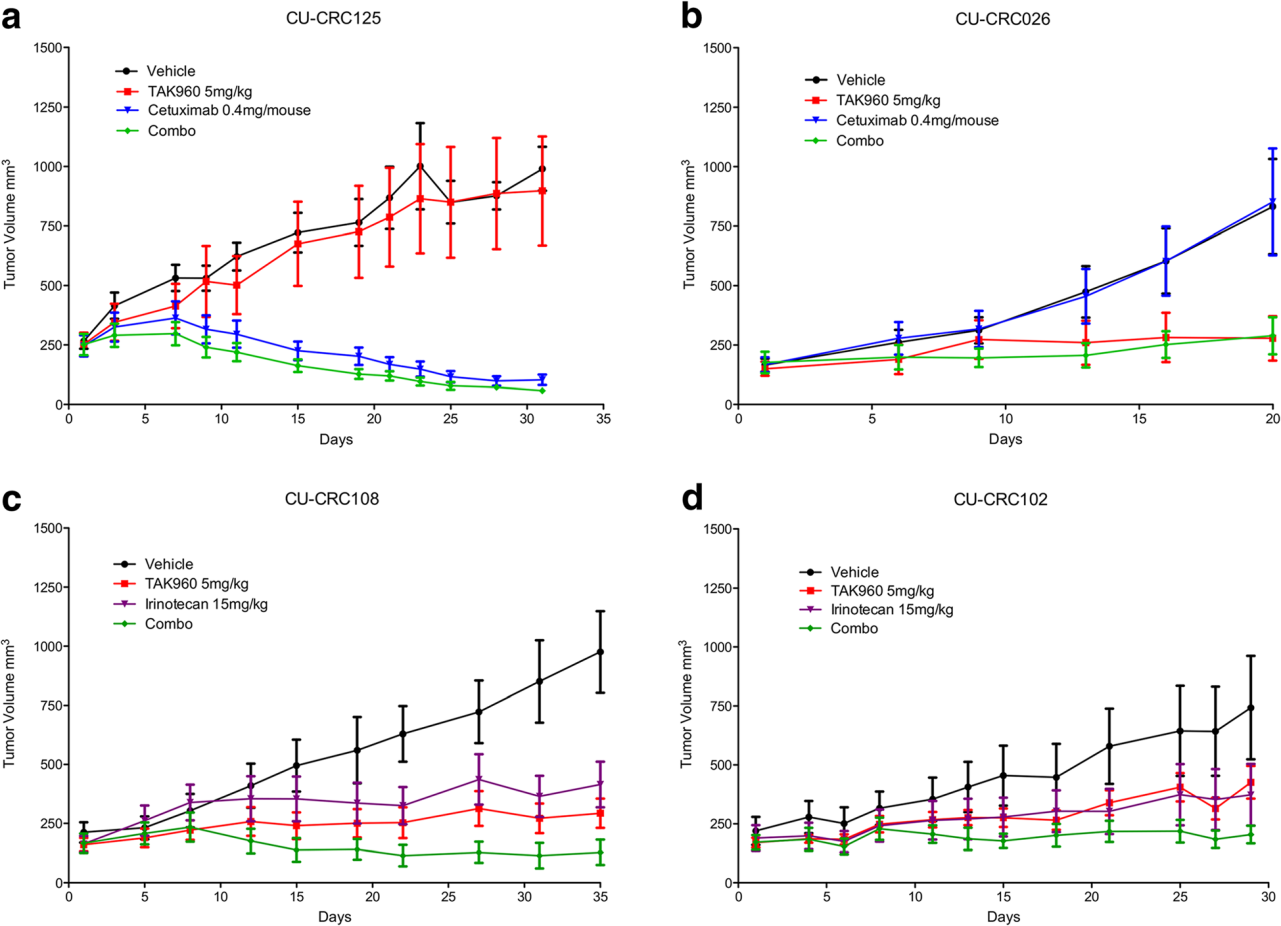
Antitumor activity of TAK-960 alone and in combination with Cetuximab or Irinotecan in CRC Patient-Derived Xenograft Models. **a**, **b** KRAS wt PDX models TAK-960 5 mg/kg + Cetuximab 0.4 mg/mouse. **c**, **d** In KRAS mut PDX models TAK-960 5 mg/kg, + Irinotecan 15 mg/kg

The combinatorial effects of TAK-960 with standard agents were evaluated in 4 PDX models (2 KRAS mutant, 2 KRAS wild type) (Fig. 6). KRAS^WT^ PDX models were treated with TAK-960, cetuximab (EGFRi) or the combination of the two. All PDX KRAS^WT^ models were treated with 10 mg/kg of TAK-960 orally once daily and 400 μg/mouse of cetuximab dosed twice per week by intraperitoneal injection. TAK-960 did not sensitize either of the 2 PDX models to cetuximab. Antitumor effects were largely driven by single-agent treatment with either cetuximab or TAK-960 (Fig. 6a, b). Because anti-EGFR therapy is not indicated for tumors with KRAS mutations, PDX models with KRAS^MUT^ were treated with TAK-960, irinotecan or the combination of the two. Although there was some evidence of an enhanced effect of the combination in both models, the results were largely additive (Fig. 6c, d).

## Discussion

Anti-mitotic therapies are a growing field in oncologic research. Plk1 is an essential gene that regulates the progression of the cell cycle through mitosis. The current study was designed to evaluate the efficacy of the investigational Plk1 inhibitor TAK-960 in colorectal cancer (CRC) models as a single agent and to determine the anti-cancer effects of TAK-960 in combination with standard agents in patient-derived CRC xenografts. The results reported here demonstrate that exposure of CRC cell lines to TAK-960 in vitro resulted in a robust anti-cancer response (31/55 cell lines had IC_50_ values < 200 nM), cytotoxicity without regrowth in colony formation assays and the induction of polyploidy. In vivo, TAK-960 therapy resulted in moderate antitumor activity (33% response rate) in our panel of 18 patient-derived colorectal tumor models, however, this response was not enhanced with the addition of standard of care agents.

Previous studies have shown TAK-960 is an effective anti-proliferative agent in in vitro models of ovarian, colorectal, sarcoma, breast, and non-small cell lung cancers, among others [18, 33, 44]. We report TAK-960 exposure has a pronounced anti-proliferative effect on CRC cell lines and IC_50_ is independent of common CRC driver mutations, including KRAS and p53 as previously reported [18].

Recent work in sarcoma cell lines shows Plk1 inhibition by TAK-960 leads to polyploidy, cell cycle arrest, and apoptosis as methods of tumor suppression. Furthermore, tumor suppression by either cell cycle arrest or apoptosis was cell line specific [33]. Consistent with these data, we observed a marked increase in polyploidy with TAK-960 treatment, however, this effect was seen in sensitive (HCT116 and WIDR) as well as resistant (DLD1) cell lines. Immunoblot experiments show the variable expression of apoptosis markers. Following TAK-960 exposure, cleaved PARP expression was not always accompanied by cleaved caspase 3. Similar findings in prostate cancer following Plk1 inhibition has suggested necroptosis as a mechanism of cellular death for some cells [45]. These data continue to muddle the concept of a binary choice between apoptosis and polyploidy. Conservatively, TAK-960 induced cell death in many models of CRC. As previously shown in TAK-960 exposure to sarcoma models, the cell fate of cycle arrest, apoptosis (possibly necroptosis) and the balance of each is largely CRC cell line specific [33].

A critical component of Plk1 inhibition as an anti-cancer strategy is its interplay with p53. Plk1 has been shown to negatively regulate p53 through transcription and protein destabilization. Likewise, Plk1 transcription is tightly regulated by p53. As p53 is mutated in over 40% of colorectal cancers, elucidating its interaction with Plk1 is valuable for treatment decisions. It is still contested whether sensitivity to Plk1 inhibition is associated with functional or non-functional p53. Early studies have demonstrated that shRNA depletion of Plk1 induces apoptosis more robustly in cancer cells with mutant or inactive p53 and that Plk1 over expression is correlated to p53 mutations [46–49]. In our studies, we found no correlation between sensitivity and p53 mutations in either the CRC cell line panel or patient derived tumor xenograft models.

It has recently been suggested that in the absence of p53, Plk1 inhibition leads to apoptosis through a p53 independent process, by which anti-apoptotic Mcl-1 is suppressed, triggering apoptosis. Furthermore, that Mcl-1 inhibition concomitant with TAK-960 can enhance the apoptotic effect [50]. While Mcl-1 levels were not evaluated in this study, it has been reported that endogenous levels of Mcl-1 are low in both HCT116 and WIDR [40]. Moreover, RKO, the most sensitive CRC cell line tested in the present study has high endogenous Mcl-1 expression. While these data do not rule out that Mcl-1 expression influences TAK-960 sensitivity or apoptotic effects, future directions should include a more thorough exploration of these conflicting data.

In the few xenograft studies that have been completed, TAK-960 has demonstrated efficacy in a wide range, but limited number of tumor types including: sarcoma, prostate, breast, lung, non-small cell lung, ovarian, myeloid leukemia and colorectal cancers [18, 33, 44]. Of these in vivo cell line xenograft experiments, only three were colorectal cancer (HCT116, HT29 and HT29) [18, 44]. In this study, we expand on these data substantially, evaluating TAK-960 therapy in eighteen additional CRC tumor models. To more faithfully recapitulate the tumor heterogeneity and architecture seen in patients, we used patient-derived xenograft rather than cell line xenograft models. Of the eighteen PDX models treated, six were classified as sensitive having a tumor growth inhibition index (TGII) less than 20%. One PDX model (CUCRC026) exhibited regression (TGII< 0).

Often, targeted therapeutics are not used clinically as a single agent. In a phase 1 trial, TAK-960 was shown to be an ineffective therapy for solid tumors when administered as a single agent (www.clinicaltrials.gov). However, Plk1 inhibition has been shown to sensitize cancer cells to gemcitabine and vincristine in vitro [51, 52]. To more faithfully replicate clinical development, we paired TAK-960 with the standard agents irinotecan and cetuximab. In the four CRC PDX models were evaluated, there was no therapeutic benefit observed in combination treatment.

Gene set enrichment analysis (GSEA) was completed on sensitive and resistant cell lines to gain insights to possible molecular vulnerabilities and combination partners to anti-Plk1 therapy. GSEA pathway analysis revealed that TAK-960 resistant cell lines exhibited increase expression of multiple cell cycle signaling nodes in series and parallel to Plk1 signaling (data not shown). While it is unsurprising that resistant cell lines would exhibit an increase in multiple alternative cell cycle signaling pathways, the extent to which global dysregulation was observed was unanticipated. These data suggest molecular inhibition of multiple targets in cell cycle pathways may be required for effective anti-cancer therapy.

Perhaps Plk1 inhibition should be rationally combined with MAP kinase pathway inhibition in TAK-960 resistant CRC. This novel combination was recently found to be robustly effective in NRAS mutated melanoma tumor models, whereas synergistic antitumor activity was observed both in vitro and in vivo [53]. These data are highly relevant to CRC, where activating mutations in the MAPK pathway (KRAS, NRAS, BRAF) occur in 50–60% of tumors [54, 55]. Moreover, studies conducted by our group and others have demonstrated that MEK inhibitors exhibit antitumor activity as single agents and in combination with other targeted therapeutics in preclinical models of CRC [56–59].

TAK-960 has been shown to be an efficacious inhibitor of proliferation in a large collection of CRC models, however mechanism of action in colorectal cancer has yet to be fully elucidated. These data suggest a potential for TAK-960 to be of therapeutic value as a single agent or in combination therapy under the right conditions. Future development of Plk1 inhibition as a therapeutic strategy for CRC will require more study into the selection of patients based upon molecular vulnerabilities, but also the development of mechanism-based rational combinations.

## Conclusions

We have demonstrated that the Plk1 inhibitor TAK-960 is a potent anti-colorectal cancer therapy through in vitro cell line assays and patient-derived tumor xenograft models. TAK-960 was shown to reduce proliferation and induce cell cycle arrest. These data form the basis of future work to elucidate rational combination partners for TAK-960, as well as strategies to select patients responsive to Plk1 inhibition.

## Additional file

Additional file 1: Table S1. IC50 values and SEM for fifty-five colorectal cancer cell lines treated with TAK-960 as assessed by CyQuant proliferation assay. (TIFF 689 kb)

## Abbreviations

ATCC: American Type Culture Collection
CRC: Colorectal cancer
DSB: Double strand breaks
ECACC: European Collection of Cell Cultures
IC_50_: Inhibitory Concentration 50%
KEGG: Kyoto Encyclopedia of Genes and Genomes
Mut: Mutant
PDX: Patient derived xenografts
PLK1: Polo-like Kinase 1
WT: wild-type
