# Supplementary figures and images for: Antitumor activity of the polo-like kinase inhibitor, TAK-960, against preclinical models of colorectal cancer
Source: BMC Cancer. 2018 Feb 5;18:136. doi: 10.1186/s12885-018-4036-z (PMC5800287; doi:10.1186/s12885-018-4036-z)

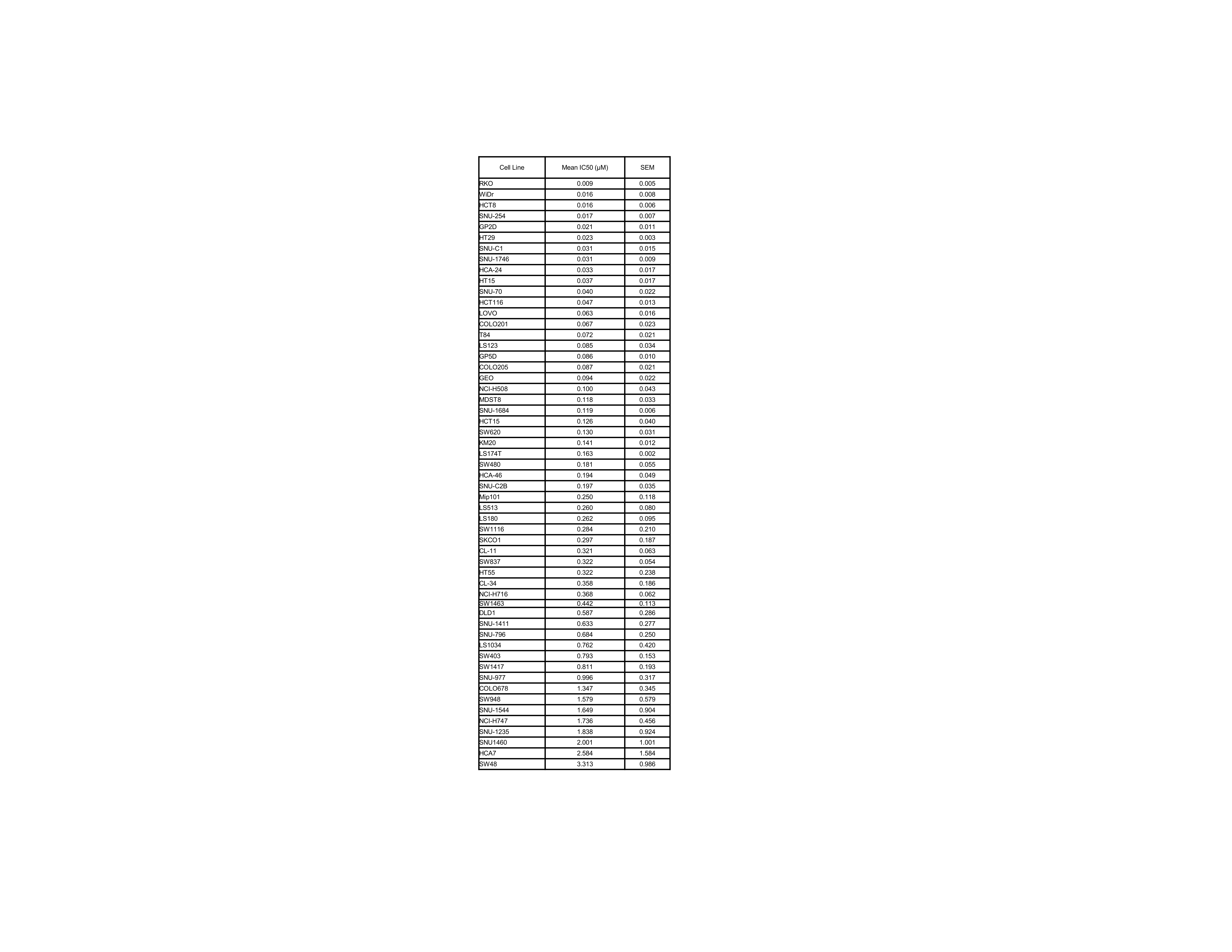

Supplement: Additional file 1: Table S1. — IC50 values and SEM for fifty-five colorectal cancer cell lines treated with TAK-960 as assessed by CyQuant proliferation assay. (TIFF 689 kb) [file 12885_2018_4036_MOESM1_ESM.tif]
